# Early Rising

**Published:** 1874-07

**Authors:** 


### EARLY RISING.

THE celebrated Dr. Doddridge mentions, in his "Family Expositor," that to his habit of early rising the world is indebted for nearly the whole of his valuable works.

Sir Thomas Moore remarks, in his preface to the "Eutopia," that he completed the work by stealing time from his sleep and meals. He made it his invariable practice to rise at four.

The well known Bishop Burnet was an habitual early riser. When at college his father aroused him to his studies every morning at four o'clock; and he continued the practice during the remainder of his life.

"I spent," says Dr. Paley, when giving an account of the early part of his life at college, "the first two years of my undergraduateship happily, but unprofitably. I was constantly in society, where we were not immoral, but idle and expensive. At the commencement of the third year, after having left the usual party at a late hour, I was awakened at five in the morning by one of my companions, who stood at my bedside, and said, 'Paley, I have been thinking what a fool you are. I could do nothing, probably, if I were to try, and I could afford the indolent life you lead. You could do everything and cannot afford it. I have had no sleep during the whole night on account of these reflections, and am now come solemnly to inform you that if you persist in your indolence I must renounce your society.' I was so struck with the visit and the visitor that I lay in bed a greater part of the day and formed my plan. I ordered my bed maker to lay my fire every evening, in order that it might be lighted by myself. I arose at five; read during the whole day; took supper at nine; went to bed; and continued the practice up to this hour." The consequence was he became a great man.

The above article reads well; and nine persons out of ten will say that it is according to reason and plain common sense that early rising is healthful, commendable, and is the guarantee of successful life. This is arrant nonsense; there is no virtue in early rising of itself. The regular whiskey drinker rises early to drink more, to satisfy his insatiable cravings; if he cannot get liquor he must have water by the quart to quench the intolerable burning within. However assiduous a man may be, and rising by daylight, he can never reach distinction if he has no brains.

The names given had intellects to begin with. The early rising was merely a concomitant, an adjunct, out of which a profitable use could be made. Paley began to rise early, but, at the same time, began to retire early. There is no virtue in rising earlier than will enable a man to get seven or eight hours of sound, refreshing sleep.

The whole fact is, that if persons go to bed early and rise betimes, and, after they have risen, employ themselves industriously, judiciously and systematically, they may reasonably expect to prosper in their respective spheres, and, if they live long enough, will rise above them.

A great deal has been said about the healthfulness of farmers, who uniformly rise early. It will be found that farmers who rise early go to bed early—generally not long after dark. But we must not mix falsehood and fact; it is a dangerous combination, constantly leading men into dangerous and fatal errors of theory and practice. It is not a fact that farmers are exceptionably healthy. The citizens of London live longer than the farming people of Great Britain, and the people of London do not think of rising earlier than eight or nine o'clock, and if they did they could see nothing but fog.

The facts narrated prove nothing. If the eminent names given rose early it is not said that they did not go to bed early. One of the most eminent men of the age became early impressed with the value of time, and was so systematic in saving the odds and ends of it, that if he happened to go into the dining room and the table was not ready, he would take a Greek testament from his pocket and read it; the same when calling on a friend, and waiting until he came into the room.

He was an early riser and retired late, *and died* in his prime, demented, in spite of an iron constitution, the author of the "Cause and Cure of Infidelity."

Quite a large number of celebrated actors and actresses have lived to a green old age, their habits compelling them to sleep until ten and twelve o'clock every forenoon for a succession of years. French women of fashion retain their good looks and health, and are often beautiful at threescore, attending balls and parties and other amusements almost every night in the week—not retiring until the small hours of the morning. One of them, on being asked how she managed, under such "dissipation," to retain her health and beauty, replied, "Because we never get up next day until we have had our sleep out." It is not known that night watchmen and temperate sea captains die earlier than other people. The fact is, that it is not getting up early or late that has a material bearing on our well being, but getting the fullest amount of sleep that the system will take. The fidgety, the nervous, the restless, the fretful, the unhappy are those who do not sleep enough; the healthy, the joyous and the blithe hearted are those who get from six to ten hours of good, sound, refreshing, heavenly sleep, every twenty-four hours. Look at childhood.

> "Early to bed and early to rise
> Makes a man healthy, wealthy and wise,"

is one of the most taking rhymes in the language, and, at the same time, is one of the most deceptive. There is nothing to recommend it unless preceded by an early retiring, and, even then, it is a positive injury the world over to all out door workers, unless they get their breakfast before they leave the house, to rise early. This early eating braces the stomach against cold and against bad air, odors and miasm.

To persons who spend most of their time in doors, what's the use of getting up hours before daylight in winter, bungling about in the dark and cold; or ruining the eyes by the sudden transition from six or eight hours of perfect darkness and disuse to the glare of artificial light and the strain of reading small print, or taking small stitches in sewing.

To get up early of a summer morning is a great trial, as all will admit, and a greater stupidity; it is perfect nonsense. No human being ever lives the longer for it, whether an out door laborer or a sedentary person, as any reader of ordinary intelligence will admit, if only one or two facts are looked at properly. No mortal man can live out his time and work mentally or physically twelve hours a day. The more work any man does the more need is there for rest in bed. Any observant man must know that he gets no refreshing sleep in the early part of the night in midsummer; it is only in the cool of the early morning that he gets real rest and refreshingness. No man can work profitably more than eight or ten hours a day; or, to put it in another form, take any dozen men and cause half of them to work ten hours a day, the other half twelve or fifteen; the ten hour men will, in every case, do more work in a week than the others, with equal hours of sleep.

It was never intended that man should work all the time in which he was neither eating nor sleeping. The Maker of the Universe "rested," and so did the Saviour of the race at the "well side." We would all live longer if we went to bed early and got up late; a rule which would prevent half the sorrows and sickness of humanity at a fair calculation. Go to bed early and keep away from the gaming table, the club room, the midnight carousal; these are the places where drunken habits are formed, where unfaithful husbands are made, and young men debauched. Going to bed early would largely tend to banish domestic infidelity and habitual drunkenness from society; and who does not know that if these things were done this earth would be a paradise to what it is now. The lessons of this article are two: First. Habitual early rising will be disastrous to mind and body unless preceded by early retiring. Second. The thrift, happiness and longevity of the race would be more promoted, if all were to remain in bed ten hours out of the twenty-four, than if only six or seven hours daily were given to sleep. Sleep is the best rest for the hard worker; the next best rest, when sleep enough cannot be had, is to remain in bed, although wide awake. It is a mischievous fallacy that it is better to be up and doing something than to remain in bed if enough sleep has not been had.

### "HE NEVER SMOKES."

This was said by a young lady, last summer, of the Emperor of Germany. She had the best opportunity of knowing, for she was a member of the Emperor William's household, near Potsdam. In addition, he retires at ten o'clock and rises at seven, giving full nine hours rest in bed—as all men over fifty ought to have, and as all hard workingmen of any age ought to have, whether they can sleep all the time or not. Rest in bed is the next best thing for recuperation ; for, let it be remembered, that when a man has become weak from labor of mind or body, he recovers from his fatigue just as an emptied mill race regains its full water power—the gate is shut down, no more water goes out, and a new supply gradually accumulates until it has a sufficient "head" to turn the machinery. Brain power, muscular power, when expended, must be regained by two processes: rest in bed, to prevent any more from going out, and then by new portions of strength coming in, which portions are supplied from the food previously eaten. But suppose no food has been taken? more or less recuperation will take place for all that; for then the system begins to use up its reserves, and, if food is not then taken, and this reserve is exhausted, then the man begins to feed upon himself, begins to consume his own flesh and blood, and he falls away until he becomes a skeleton; this is nature's method of sustaining life under the accidents of forced deprivation of food, as in shipwrecks, being lost in desert places, and the like. When Franklin connected "early rising" with health, wealth, and all that, he meant, of course, that the time gained by early rising should be advantageously employed. The true sense would be more fully expressed by adding another line to the homely rhyme—

> "Early to bed and early to rise,
> Makes a man healthy, wealthy and wise,"
> If he will freely advertise.

You must do something else besides getting up early if you want to become successful in life. A man, besides having a good store or shop, must let the people know where that shop is and what is in it, otherwise he will have but few calls for his wares or his work. It is almost the universal habit of the old nations of the world to rise late; this would seem to have been the dictate of ages of experience and observation; the common sense of the masses appears to have adopted the custom as the best, under the circumstances. At the same time we have no objection to a man getting up yesterday, if he wants to. But let any man try the experiment for one week of sleeping seven hours and resting in bed two hours more, and see if he will not have done more work by Saturday night, and have a less fagged out feeling, than if he were in bed only seven hours, and added the extra two to his usual day's labor. We must, in these things, experiment more and reason less—depend more on trials than on theory.

After all, facts are most convincing. Last spring a very thriftless fellow thought he would turn over a new leaf and embark in a new enterprise "by times." So he got up at daylight, went over to his neighbor's crib to get some seed corn; before he had filled his bag a dog bit him on the heel, whereby he was confined to his house for seven months. As soon as he got well, being very persevering, he rose before the sun and paid a visit to a watermelon patch, and, while he was admiring one of the largest specimens, a bit of lead came along and shaved off one of his ears. Now, it is easy to see, if that man had only stayed in bed two or three hours longer the dog would not have bitten him, and that piece of lead would have missed him entirely. It seems to be plain, then, that, to be benefited by early rising, a man should go to bed early, and, when he gets up, should busy himself in some legitimate and useful employment.

